# Attention Deficit/Hyperactivity Disorder (ADHD) Diagnosis: An Activation-Executive Model

**DOI:** 10.3389/fpsyg.2016.01406

**Published:** 2016-09-21

**Authors:** Celestino Rodríguez, Paloma González-Castro, Marisol Cueli, Debora Areces, Julio A. González-Pienda

**Affiliations:** Department of Psychology, Faculty of Psychology, University of OviedoOviedo, Spain

**Keywords:** activation, execution, ADHD, diagnosis, blood-flow oxygenation, structural equation modeling

## Abstract

Attention deficit with, or without, hyperactivity and impulsivity (ADHD) is categorized as neuro-developmental disorder. ADHD is a common disorder in childhood and one of the most frequent conditions affecting school ages. This disorder is characterized by a persistent behavioral pattern associated with inattention, over-activity (or hyperactivity), and difficulty in controlling impulses. Current research suggests the existence of certain patterns of cortical activation and executive control, which could more objectively identify ADHD. Through the use of a risk and resilience model, this research aimed to analyze the interaction between brain activation variables (nirHEG and Q-EEG) and executive variables (Continuous performance test -CPT-) in subjects with ADHD. The study involved 499 children, 175 females (35.1%) and 324 males (64.91%); aged from 6 to 16 years (*M* = 11.22, *SD* = 1.43). Two hundred and fifty six of the children had been diagnosed with Attention Deficit Hyperactivity Disorder (ADHD) and 243 were without ADHD. For the analysis of this objective, a causal model was designed to include the following different measures of task-execution: CPT TOVA (omissions, commissions, response time, variability, D prime and the ADHD Index); electrical activity (using Q-EEG); and blood-flow oxygenation activity (using nirHEG). The causal model was tested by means of structural equation modeling (SEM). The model that had been constructed was based upon three general assumptions: (1) There are different causal models for children with ADHD and those without ADHD; (2) The activation measures influence students’ executive performance; and (3) There are measurable structural differences between the ADHD and control group models (executive and activation). In general, the results showed that: (a) activation measures influence executive patterns differently, (b) the relationship between activation variables (nirHEG and Q-EEG) depends on the brain zone being studied, (c) both groups showed important differences in variables correlation, with a good fit in each model (with and without ADHD). Lastly, the results were analyzed with a view to the diagnosis procedure. Therefore, we discuss the implications for future research.

## Introduction

Attention deficit with, or without, hyperactivity and impulsivity (ADHD) is one of the disorders that most affects academic performance. Current research suggests the existence of certain patterns of cortical activation and executive control, which could more objectively identify ADHD. To detect these patterns, brain activation variables are recorded in the areas of central and prefrontal cortex through electro-encephalographic techniques such as quantified EEG (Q-EEG) to measure beta-theta electrical activity ratios (González-Castro et al., 2013), as well as oxygenated blood-flow in the brain (hemo-encephalography: nirHEG) (Toomim et al., 2005; Toomim and Carmen, 2009). In addition, executive control is evaluated with tests to verify levels of cortical activation by measuring performance during a lengthy repetitive task known as the Continuous Performance Test (CPT).

On the other hand, with the publication of the new DSM-5 classification manual (American Psychiatric and Association [APA], 2013), ADHD is now categorized as neuro-developmental disorder. While there were no significant changes in terms of the main symptoms of the disorder, with respect to classification there are now three types of presentations (instead of subtypes) of ADHD: predominantly hyperactive/impulsive; predominantly inattentive; and combined presentation. However, regardless of the names used for classification, much research has investigated if ADHD subtypes (or types of presentation) differ in their development or in their epidemiology (Willcutt et al., 2012), and also whether different comorbidities generally associated with the disorders are dependent upon the subtype (Sciberras et al., 2014).

### ADHD, Cortical Activation and Execution

Although there is a substantial body of symptom-based evidence highlighting the neurologic nature the disorder, the primary causal factors underlying this problem remain unclear to date (Rubia et al., 2011; Tsujimoto et al., 2013; Congdon et al., 2014).

Within this context, some investigations point to a delay in myelination formation during brain development (Sowell et al., 2003), or insufficient white matter in the frontal lobe (Mostofsky et al., 2002). A further potential factor may be early dysfunctions in executive functions associated with fronto-thalamic circuits (Brown, 2006), which have a direct impact on cortical activation levels (Lubar et al., 1995; Álvarez et al., 2008; Cortese et al., 2012; Orinstein and Stevens, 2014).

From a general perspective, ADHD has been associated with a dysfunction in the central nervous system, characterized by a developmental delay and cortical hypo-activation related to a deficit in the dopaminergic and noradrenergic systems (Bledsoe et al., 2011). The noradrenergic system is primarily responsible for the modulation of selective attention and the levels of general activation that an individual needs to perform a task (Brown, 2006). The dopaminergic system, in turn, is associated with the ability to control one’s behavior, both at an executive and motivational level. Thus, this low cortical activation associated with dopaminergic and noradrenergic systems would at least partially explain the inhibitory and attentional deficits that characterize ADHD (Cubillo et al., 2012). Furthermore, the investigation of González-Castro et al. (2013) showed that the low activation in prefrontal areas was reflected in different patterns of executive control measured in a CPT.

The above hypothesis is supported by neo-connectionist learning models, which have also linked cortical activation (measured by means of frequency fields) with the cortical areas involved in ADHD (Congedo and Lubar, 2003; Orlando and Rivera, 2004; Mazaheri et al., 2014; Orinstein and Stevens, 2014). When the subject is distracted, frequency fields are characterized by delta or theta waves, with a frequency of 0.5–4 Hz and 4–8 Hz, respectively. When the subject is relaxed with scattered attention, brain theta waves have values between 8 and 12 Hz. Finally, when the subject is in an alert state, beta waves with frequency ranges from 15 to 35 Hz are dominant. These waves are produced by brain metabolism and blood flow, as shown by Lubar et al. (1995). In this sense, an increase in theta activity would be accompanied by decreases in blood flow and brain metabolism. Hence, high frequencies of theta activity are commonly observed in brain areas with low activation (Álvarez et al., 2008).

Concerning ADHD, a differential pattern of electro-cortical activity has been observed in a state of rest, and it is characterized by increased theta -and decreased beta- activity (Lansbergen et al., 2011). This profile has been reflected in different studies with a low cortical activity associated with decreased beta activity in central and prefrontal brain regions in students with ADHD (Ernst et al., 2003). The detection of this pattern of cortical hypo-activation has been made using different neuro-imaging techniques, such as functional magnetic resonance imaging (fMRI) (Logothetis and Wandell, 2004; Solanto et al., 2009), electro-encephalography (EEG) (Mazaheri et al., 2014), or hemo-encephalography (HEG) (Schecklmann et al., 2009).

On the other hand, increasing cortical activation has been observed in students with ADHD who have had positive responses to intervention, and this has led to a decrease in inattention, impulsivity and hyperactivity according to previous research (Monastra et al., 2005; Kropotov et al., 2007; Arns et al., 2009). For example, a study conducted by Thompson and Thompson (1998) involving 111 subjects (children and adults) with ADHD observed significant improvements in cortical activation (measured by Q-EEG) and symptomatology (measured by CPT), following an intervention involving neurofeedback techniques.

Other studies have also found that, by increasing cortical activation with neurofeedback techniques or pharmacological support, individuals with ADHD significantly improved their performance in attention tasks, apparently as a consequence of a decrease in the core symptoms of the disorder (Othmer et al., 2000; Fuchs et al., 2003; Rossiter, 2004). Also, Monastra et al. (2005), in a review, analyzed the empirical evidence of the intervention with neurofeedback, according to the Association of Applied Psychophysiology and Biofeedback and the International Society for Neuronal Regulation. They concluded that neurofeedback is “probably an efficacious instrument” for treatment of ADHD, as clinically significant improvement is observed in approximately 75% of the cases analyzed.

In sum, previous research supports the relationship between ADHD symptoms and decreased cortical activation. Nevertheless, although it has been argued that low activation occurs in prefrontal and frontal areas, the specific areas involved in these processes have not been adequately defined (Orinstein and Stevens, 2014). The most frequently reported areas in this case have been in the pre-frontal (e.g., Fp1, Fp2, Fp3) and central (e.g., Cz) regions (Hale et al., 2007; González-Castro et al., 2013).

The difficulties in the detection of specific brain areas have been associated with the presence of differential profiles or presentations in the disorder (Nikolas and Burt, 2010; Willcutt et al., 2012). Thus, the relevance of these areas would be dependent on the presence of inattentive or hyperactive/impulsive symptomatology (Depue et al., 2010; Mazaheri et al., 2014). Considering the different presentations of ADHD, previous studies have shown that while the hyperactive/impulsive presentation is related to poor activation in left prefrontal areas, the inattentive presentation is commonly accompanied by less activation in central and central-prefrontal areas (González-Castro et al., 2013). Similarly, it has been observed that students with low levels of activation in left prefrontal areas show more commission errors and higher variability in CPTs, while students with low central activation show more omissions and slower response time than the other group.

The empirical evidence concerning the different categories of symptomatology in ADHD, and their new conceptualization in DSM-5 (American Psychiatric and Association [APA], 2013), makes it necessary to define the relationship among the levels of activation in specific brain areas, executive functions, and diagnosis-related variables (i.e., distinction between ADHD and controls, and among different ADHD presentations).

It is important to consider that this disorder not only leads to impairments in the academic context (Frazier et al., 2007; Barnard-Brak et al., 2011), but also in the social and familiar contexts (Anastopoulos et al., 2009; Schroeder and Kelley, 2009). It is therefore crucial to have appropriate evaluation strategies that are able to minimize error in the diagnosis process (Skounti et al., 2007). This particular aspect was the key stimulus for the present study. Although the exact cause of the disease has not yet been identified, it is thought to be caused by a complex interaction between the neuro-anatomical system and neuro-biochemistry rather than a single cause. Overall, an increased number of findings suggest that ADHD is a disease of the brain (Swanson and Castellanos, 2002). Thus, genetic factors, neuro-developmental factors, psychosocial factors, and neuro-physiological factors all have an influence on behavior, activity and task-execution.

By using a risk and resilience model, this research aims to analyze the interaction between brain activation variables and executive function in students with ADHD. For the analysis of this objective, a causal model (relationship between pre-frontal cortex activation and task-execution) was formulated in which different measures were included (CPT-TOVA, Q-EEG and nirHEG; Toomim et al., 2005).

### Purposes of This Study

By means of a structured equation model (SEM) we expect to deepen our knowledge of the relationship between activation measures and executive function measures. The SEM designed was fit using two samples of data (control group without ADHD and ADHD group). The first sample (without ADHD) was utilized to fit the model, and the second sample (with ADHD) to analyze the consistency of the data with predictive differences. We also performed multi-group analysis to verify the consistency of the results from both samples, to know which variables differentiate subjects with and without ADHD.

**FIGURE 1:**
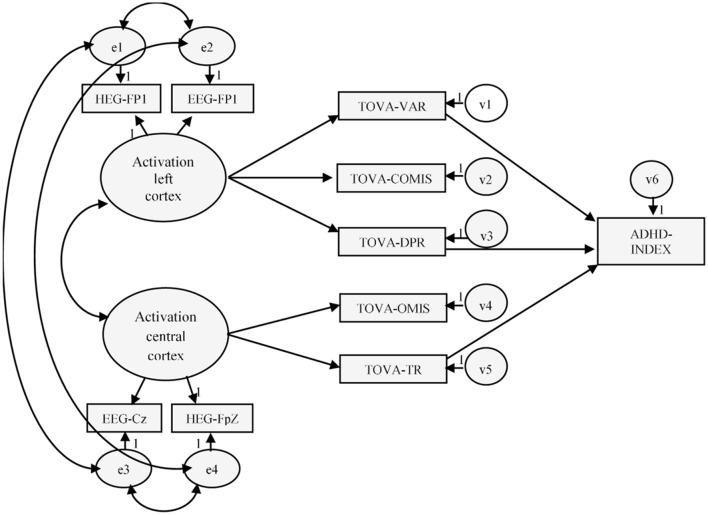
**Hypothetical model of activation and executive function**. Variables in the model: HEG-Fp1 (nirHEG ratio: left pre-frontal cortex); HEG-FpZ (nirHEG ratio: central zone pre-frontal cortex); Q-EEG-Fp1 (beta-theta ratio: left pre-frontal cortex); Q-EEG-CZ (beta-theta ratio: central cortex); TOVA-OMIS (CPT: omissions); TOVA-COMIS (CPT commissions); TOVA-VAR (CPT: variability); TOVA-TR (CPT: response time); TOVA-DPR (CPT: D prime); ADHD-INDEX (CPT: ADHD index).

Considering the data provided by literature findings, the causal model was tested using structural equation modeling (SEM). This model was built based on three general assumptions (see **Figure 1**):

(1) There are different causal models for children with ADHD and those without ADHD.

(2) The activation measures influence a student’s executive performance. Specifically, certain task-execution variables will be related to activation in the left pre-frontal cortex, and others with central zone pre-frontal cortex activation.

(3) There are important structural differences between the models for the ADHD and control groups.

When estimating the dependent variables of the model (latent variables), we also assume that the measured errors are not inter-correlated in the model, and that there is no relationship between the types of errors committed. Lastly, although previous research indicates reciprocal relationships among the dependent variables measured in this model (omissions, commission, and response time -RT-, variability and D prime), in the current investigation it is theoretically unacceptable to expect that reciprocal relationships between causal measures have been observed at a single temporal moment.

Our model has two parts: one of measurement, which corresponds to the relationship between the latent variables and their respective observed variables (activation), and a structural part, which involves the relationship between the independent and the dependent variables of the model (execution). The effects of the independent on the dependent variables are indicated with gamma (γ), whereas the relationships among the dependent variables are represented as beta (β).

## Materials and Methods

### Participants

The participants included in the study comprised 499 students aged between 8 and 16 years (*M* = 11.22, *SD* = 1.43). There were 324 males (64.9%) and 175 females (35.1%). As one of the goals of this research was the cross-validation of the study-model developed, the final calibration sample was split into two subgroups [243 (48.7%) in the Control Group, and 256 (51.3%) in the ADHD group]. All participants had an IQ higher than 80 (WISC-IV; Wechsler, 2005), were attending public and subsidized schools in northern Spain. Statistical analysis revealed no significant between-group differences concerning IQ, though there were slight differences in mean ages and gender ratios (**Table 1**).

**Table 1:** Means (M) and standard deviations (SD) of IQ scores, age in months, and EDAH percentile scores of the two groups in the sample (Control and ADHD group).

		Control group	ADHD group	*Total sample*	
*N*		243	256	499	
IQ *M (SD)*		98.30 (10.28)	98.95 (10.15)	98.64 (10.21)	*F*(1,497) = 0.496, *p* = 0.481, η^2^ = 0.001
*Age (months) M (SD)*		136.67 (17.51)	132.88 (16.77)	134.72 (17.22)	*F*(1,497) = 6.102, *p* = 0.014, η^2^ = 0.012
*Sex (male/female)*		146/97	178/78	324/175	χ^2^ (1) = 4.888, *p* = 0.027
*EDAH scores*	*ADHD-I*	73.84 (10.71)	90.96 (5.44)	82.62 (12.01)	*F*(1,497) = 514.33, *p* = 0.000, η^2^ = 0.509
	*ADHD-HI*	74.49 (10.59)	92.05 (5.20)	83.50 (12.06)	*F*(1,497) = 561.34, *p* = 0.000, η^2^ = 0.530
	*ADHD-C*	75.77 (9.90)	91.46 (6.17)	83.82 (11.34)	*F*(1,497) = 456.27, *p* = 0.000, η^2^ = 0.479

*(ADHD-I) subtype with predominance of attention deficit; (ADHD-HI) subtype with predominance of hyperactivity–impulsivity; and (ADHD-C) combined subtype, with predominance both of inattention and hyperactivity-impulsivity*.

#### Inclusion Criteria

For ADHD the diagnosis involved: (a) clinical diagnosis of Attention Deficit Disorder with Hyperactivity according to the Diagnostic and Statistical Manual of Mental Disorders-IV-R (American Psychiatric and Association [APA], 2002); (b) symptom duration of more than 1 year; (c) the problem began before the age of 7 years; and, (d) the children had no associated disorders. Subjects who presented with a cognitive deficit, Asperger’s syndrome, Guilles de la Tourette syndrome or extensive anxious depressive disorders were excluded from the study, (e) to confirm the diagnosis and rule out other associated disorders, all students underwent a semi-structured interview for parents Diagnostic Interview Schedule for Children DISC-IV (Shaffer et al., 2000), and (f) were administered the WISC-IV (Wechsler Intelligence Scale for Children-IV; Wechsler, 2005) to evaluate the presence of specific (or other) cognitive deficits.

All healthy controls underwent the same diagnostic assessment to rule out any psychiatric disorders. To ensure the correct assignment of the students to their respective groups, Farré and Narbona’s (1997) Spanish Scale or the adaptation by Sánchez et al. (2010) for ADHD (EDAH) was administered to the participants’ parents.

### Instruments and Measures

The variables included in the hypothesized model were grouped into two categories: activation measures (nirHEG Fp1, nirHEG FpZ, Q-EEG Fp1 and Q-EEG Cz), and executive measures (omissions, commissions, variability, RT, D prime and ADHD Index).

#### Activation Measures

*The nirHEG* (Toomim et al., 2005) is a tool used to measure blood oxygenation in expressly selected areas. The nirHEG employs the translucent properties of biological tissue, and low-frequency red and infrared light from light emitting diodes (LEDs). The source of light and the light receptor (optode) are mounted on a headband 3 cm apart. The band should be carefully placed so that no external light enters. It is important to highlight that, in contrast with the EEG method, low muscular tension or small subject movements do not affect nirHEG measurements. Other possible sources of error were researched and were found to be minimal (Toomim et al., 2005). Only around 5–10% of nirHEG readings come from the skull skin or tissue because these regions of the body have little blood flow in comparison with brain tissue. The depth of effective penetration in the highly vascular cortical tissue is approximately 1.5 cm below the midpoint between the light source and the receptor optode. The entrance and exit light areas are 0.052 cm^2^ at the skin surface. The light entrance and exit points and the refractive and scattering qualities of the tissue form a banana-shaped light field.

The lights are emitted alternately onto the surface of the skin. The emitted light penetrates these tissues and is scattered, refracted, and reflected. A small amount of light modified by absorption of the tissue returns to the surface and is measured. The ratio is calculated by comparing the red light (at 660 nm wavelength), which is not absorbed as much by oxygenated hemoglobin, with infrared light (at 850 nm wavelength), which is less affected by oxygenation (Toomim et al., 2005). Capillary oxygenation is barely affected by peripheral blood pressure and is mainly controlled by tissue demand for energy. The concentration of oxygenated hemoglobin is therefore a useful measurement of local blood flow. Thus, mathematically, the formula for the nirHEG ratio is as follows: nirHEG Ratio = Red light (variable)/infrared light (less affected by oxygenation).

The nirHEG Ratio or proportion between red and infrared light has a useful property. The numerator and denominator in the relationship are influenced in the same way by attenuation of the skin, the skull, and the length of the path. In this relationship, these variables are therefore discarded. The standardized reference value was established at 100 (*SD* = 20) and used to calibrate all new spectrophotometers (Toomim et al., 2005).

In addition to this measure, nirHEG provides an Attention Index (AI), indicating malfunctioning of the ability to increase the nirHEG ratio; that is, the participant is incapable of increasing the ratio and, thereby, brain activation. This apparently indicates a lapse in the attentional process, which, according to Toomim et al. (2005), is equivalent to a measure of sustained attention or concentration capacity.

*Q-EEG (quantified electroencephalogram)*, Biocomp 2010 (Developed by The Biofeedback Institute of Los Angeles^1^) was used to record electrical activity. Q-EEG (quantified electroencephalogram) is a computerized EEG system, adapted by Toomim et al. (2005), which provides levels of cortical activation through the beta/theta ratio. It measures attention in general, independently of the task to be performed. For this purpose, an electrode is placed on the subject’s corresponding cortical area (Cz, Fp1) to record the beta/theta ratio, and two more control electrodes are placed on the subject’s left and right earlobe. The Q-EEG is administered to each participant, with open eyes, for a maximum duration of 10 min and after receiving instructions of smooth and steady abdominal breathing, in order to carry out the test under the best possible performance conditions. Lastly, an EMG system is placed on the right forearm to identify the degree of movement. Once the electrodes are in place, participants are asked to remain relaxed, without moving, breathing slowly and evenly, concentrating exclusively on the computer screen on which the theta and beta waves emitted by them are displayed successively. After assessment, the results obtained are interpreted. When the beta/theta ratio is lower than 50% at Cz, there is an associated deficit of sustained attention and if the ratio is also lower at Fp1, then the attentional deficit is associated with a lack of executive control, attributable to hyperactivity (González-Castro et al., 2013).

#### Latent Variables (Pre-frontal Cortex Activation)

*Activation left cortex* was estimated as a latent variable in the SEM from two indicators of activation measures. One of the indicators was nirHEG in Fp1 and the other was Q-EEG in Fp1. Thus, our latent variable takes into account the commonalities between these two ratio-index measures of the of the student’s cortical activation.

*Activation central cortex* was estimated as a latent variable. One of the indicators was nirHEG in FpZ and the other was Q-EEG in Cz. So, our latent variable subsumes the communalities between this two ratio measures indexes of the students’ activation.

#### Executive Functioning Variables

*Test of Variables of Attention* (TOVA; Greenberg and Waldman, 1993) is a CPT that presents two simple images. The first one presents the stimulus at the top of the screen and the second one at the bottom of the screen. The subject is given a push-button that should only be pressed when the first image appears. Subjects are trained for 3 min before testing, and the test lasts between 20 and 24 min. The following profile is obtained: omissions, RT, commissions, variability, D prime (performance and/or concentration quality during the test, based on the number of errors) and ADHD Index. In the current study, the Cronbach’s alpha for this executive factor was 0.877.

### Procedure

The identification of the participants was carried out according to the DSM-IV-TR criteria in the Hospital Pediatric Service by a neurologist with experience in ADHD diagnosis. It was confirmed by the EDAH with parent–teacher agreement equal to or higher than 90% following previous studies (González-Castro et al., 2015). Once the ADHD group was established, we proceeded to select the students who made up the group without ADHD, so that the groups would be as equivalent as possible. For this purpose, all the participants completed the WISC-IV (Wechsler, 2005), and their age was also taken into account. Once identified, if their IQ was equal or higher than 80, they completed the TOVA. Both tests (WISC-IV and TOVA) were interpreted according to their corresponding instruction manuals. Participants were not undergoing pharmacological treatment during the study. It was withdrawn 48 h to perform the tests.

After psychological assessment and the appraisal of executive control, the level of cortical activation was identified by means of the Q-EEG analysis, using the Biocomp 2010. The surface electrodes were placed at points Fp1 and Cz. To control participants’ movement, an Electromyogram (EMG) electrode was placed on the right fore-arm and the reference electrodes were placed on the ear lobes. The recording was carried out in a sound-proof and electrically isolated room with low illumination, and the test always at the same time of day (between 4 p.m. and 6 p.m). The Q-EEG was administered to each participant (with their eyes open), and for a maximum duration of 10 min. The nirHEG was administered in the same circumstances of q-EEG. With a measurement of 35 seconds in Fp1 and FpZ duly counterbalancing the order with the characteristics of the band measurement described above. The TOVA measures were standardized, interpreting scores lower than 1.2 standard deviations as negative measures. Lastly, a general executive control index showing recorded readings lower than -1.80 was interpreted as ADHD. For the partial correlations, we took age into account because activation and executive control both tend to decrease with age.

The study was conducted in accordance with The Code of Ethics of the World Medical Association (Declaration of Helsinki), which reflects the ethical principles for research involving humans (Williams, 2008). All subjects and their parents gave written informed consent after receiving a comprehensive description of the study protocol. Participants had volunteered to be involved in this study and they were not given any incentive to take part in it. The participants came from families of medium socio-economic status and were Caucasian

### Data Analysis

The adequacy of the model was analyzed with SEM, using the AMOS.22 program (Arbuckle, 2009). Firstly, the data matrix (control group and ADHD group samples) was analyzed to determine whether there were any values that violated any of the assumptions required for the use of SEM (e.g., multivariate normality, linear relations among variables, absence of multi-collinearity), or simply whether there were any missing data or outliers. Subsequently, the fit of the model was examined utilizing the control group sample and, although the hypothesized model fitted well, potential areas of misfit in the model were scrutinized (by examining the standardized residuals and the modification indexes). Secondly, we followed an invariance-testing strategy to test the structural paths across groups to determine whether the models of the Control Group and of the ADHD Group were equivalent. In order to cross-validate our data-analysis, we fitted the model to an independent clinical sample of students (the ADHD sample).

## Results

### Initial Data Screening

**Table 1** shows the descriptive data as well as the two Pearson correlations matrixes corresponding to the Control Group and the ADHD group. Before conducting the statistical analyses, we examined the matrixes with regard to missing data, the presence of outliers, linearity and normality of the data. We examined the data to determine whether any of the variables or subjects presented a significant amount of missing values. Considering the variables with respect to Kline’s (2013) suggestions, the number of absences was found to be less than 1.4% in all cases, which was not significant.

One of the important assumptions of SEM is that the variables taken must follow a normal distribution. As maximum likelihood (ML) can produce biases when this assumption is violated (West et al., 1995), we examined the distribution of the variables (i.e., kurtosis and skewness). Following the criteria of Finney and DiStefano (2006), the allowable values for skewness and kurtosis are ±2 and ±7 respectively (outside of which, ML should not be used). All the variables in this study respected those criteria (see **Table 2**). Therefore, with normality conditions being met, we decided to fit the model using ML.

**Table 2:** Correlation matrix corresponding to the variables included in the model (Control group and ADHD group) and descriptive data (means, standard deviation, skewness and kurtosis).

	1	2	3	4	5	6	7	8	9	10
1	-	0.499^∗∗^	0.594^∗∗^	0.330^∗∗^	0.306^∗∗^	0.435^∗∗^	0.514^∗∗^	0.213^∗∗^	0.213^∗∗^	0.222^∗∗^
2	0.441^∗∗^	-	0.315^∗∗^	0.743^∗∗^	0.471^∗∗^	0.258^∗∗^	0.304^∗∗^	0.366^∗^	0.183^∗^	0.218^∗∗^
3	0.848^∗∗^	0.303^∗∗^	-	0.376^∗∗^	0.238^∗∗^	0.290^∗∗^	0.330^∗∗^	0.125	0.108	0.090
4	0.428^∗∗^	0.842^∗∗^	0.387^∗∗^	-	0.447^∗∗^	0.193^∗∗^	0.203^∗∗^	0.380^∗∗^	0.122	0.159^∗^
5	0.456^∗∗^	0.757^∗∗^	0.371^∗∗^	0.795^∗∗^	-	0.342^∗∗^	0.449^∗∗^	0.667^∗∗^	0.389^∗∗^	0.447^∗∗^
6	0.720^∗∗^	0.378^∗∗^	0.660^∗∗^	0.398^∗∗^	0.453^∗∗^	-	0.505^∗∗^	0.132^∗^	0.428^∗∗^	0.358^∗∗^
7	0.811^∗∗^	0.309^∗∗^	0.816^∗∗^	0.356^∗∗^	0.425^∗∗^	0.722^∗∗^	-	0.428^∗∗^	0.380^∗∗^	0.458^∗∗^
8	0.411^∗∗^	0.731^∗∗^	0.339^∗∗^	0.753^∗∗^	0.852^∗∗^	0.441^∗∗^	0.409^∗∗^	-	0.339^∗∗^	0.479^∗∗^
9	0.698^∗∗^	0.475^∗∗^	0.703^∗∗^	0.559^∗∗^	0.571^∗∗^	0.678^∗∗^	0.725^∗∗^	0.525^∗∗^	-	0.813^∗∗^
10	0.644^∗∗^	0.492^∗∗^	0.670^∗∗^	0.580^∗∗^	0.631^∗∗^	0.614^∗∗^	0.750^∗∗^	0.596^∗∗^	0.874^∗∗^	-
**Control group**										
*M*	101.64	105.50	0.58	0.59	98.76	100.90	97.44	99.37	0.49	1.51
*SD*	12.40	17.45	0.07	0.07	8.01	10.09	8.65	10.45	1.05	2.29
Skewness	0.970	1.192	1.181	0.909	0.216	0.097	0.708	0.506	0.572	0.461
Kurtosis	0.926	1.158	4.314	1.331	-0.150	1.024	0.913	1.839	-0.089	-0.238
**ADHD group**										
*M*	78.52	79.82	0.43	0.43	77.05	82.83	76.55	77.67	-1.49	-3.39
*SD*	10.71	12.04	0.07	0.07	10.82	10.82	10.13	10.06	0.89	1.89
Skewness	0.501	1.138	-0.070	-0.033	-0.017	-0.052	-0.058	0.528	-0.207	-0.548
Kurtosis	2.399	4.048	0.118	0.457	1.469	1.321	0.544	2.702	0.008	-0.097

*In the correlation matrix, the upper matrix corresponds to the without ADHD sample and the lower matrix to the ADHD group sample. 1, nirHEG-Fp1; 2, nirHEG-FpZ; 3, Q-EEG-Fp1; 4, Q-EEG-CZ; 5, TOVA omissions; 6, TOVA commissions; 7, TOVA variability; 8, TOVA response time; 9, TOVA D prime; 10, TOVA ADHD index*.

*^∗^*p* < 0.05; ^∗∗^*p* < 0.001*.

Another important aspect in the initial analysis of the data matrix is to verify that the variables are significantly correlated, although such correlations should not be excessively high (*r* > 0.85). The pattern of correlations (e.g., size; + tendency) was similar both groups.

### Testing and Adjusting Model (Control Group)

In a first assessment of the model (**Figure 1**), the estimated parameters did not show the expected magnitudes and mathematical sign (consistent with the theory underlying the model), and excessive standard errors were observed (Bentler, 1995). The data provided by the analyses performed with AMOS.22 indicated that the fit of the hypothesized model to the data matrix was not acceptable, χ^2^(28) = 81.11, χ^2^/df = 2.89, *p* < 0.001, GFI = 0.939, AGFI = 0.881, TLI = 0.928, CFI = 0.928, RMSEA = 0.089 (0.066–0.111), *p* = 0.003.

#### Re-specification of the Model

After examining the residuals and modification index (although the hypothesized model did not show a good fit), we considered the possibility of including covariance effect between Commissions and RT in the TOVA test (leaving the parameter free) as well as the indirect effect contained in the initially hypothesized model. At the theoretical level, this effect is negative, indicating that a higher number of commissions the response time will be less in TOVA.

**FIGURE 2:**
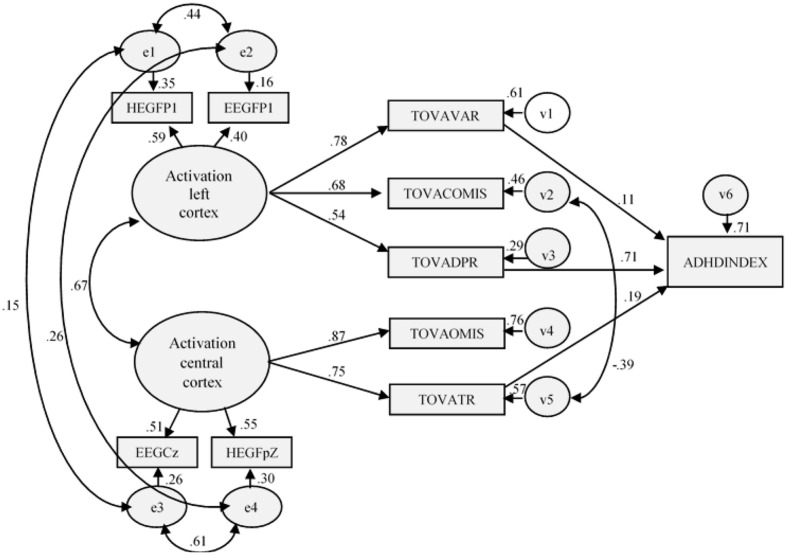
**Re-specified model (Control Group): structural and measurement coefficients**.

The results indicated that the fit of the re-specified model was good, [χ^2^(27) = 57.924; χ^2^/df = 2.145; *p* ≤ 0.001; GFI = 0.954; AGFI = 0.907; CFI = 0.974; TLI = 0.956; RMSEA = 0.069 (0.044–0.093), *p* = 0.098], and the improvement over the initial model was statistically significant (Δχ^2^(1) = 23.192). As expected, the new estimated parameter was statistically significant and negative (*r* = -0.39). Neither the residuals nor the modification indices recommended carrying out more changes in the model (**Figure 2**). **Table 3** shows the coefficients of the relationships in the measurement model and the structural model, as well as their corresponding estimation errors, critical ratios, and associated probabilities.

**Table 3:** Results of testing the re-specified model (sample without ADHD).

	Standardized Coefficients	*SE*^1^	CR^2^	*P*<^3^
**Structural Model^4^**				
Activation left cortex → TOVA variability	0.783	0.114	8.308	0.001
Activation left cortex → TOVA D prime	0.537	0.012	6.601	0.001
Activation central cortex → TOVA response time	0.753	0.102	8.041	0.001
Activation left cortex → TOVA Commissions	0.678	0.125	7.687	0.001
Activation central cortex → TOVA Omissions	0.870	0.088	8.337	0.001
TOVA D prime → TOVA ADHD Index	0.712	0.083	18.539	0.001
TOVA variability → TOVA ADHD Index	0.108	0.011	2.689	0.007
TOVA response time → TOVA ADHD Index	0.193	0.008	5.104	0.001
***Measurement Model^5^***				
Activation left cortex → nirHEG-Fp1	0.589	-	-	-
Activation left cortex → Q-EEG-Fp1	0.399	0.001	6.785	0.000
Activation central cortex → nirHEG-FpZ	0.552	-	-	-
Activation central cortex → Q-EEG-Cz	0.511	0.000	10.371	0.000

^1^*Standardized errors,*^2^*Critical ratio,*^3^*Probability,*^4^*structural model (relation between the independent and the dependent variables in the model)*, ^5^*measurement model (relation between the latent variables in the model and the observed variables)*.

With regard to the assessment of the predictions implicit in the re-specified model without ADHD, the results indicated that almost all hypotheses were confirmed in measurement part. Latent variable named *Activation left cortex* was significantly and positively explained by Q-EEG-Fp1 (β = 0.40), however, in contrast to our prediction, its relation with nirHEG-Fp1 (β = 0.59) was not statistically significant. Activation central cortex was significantly and positively explained by Q-EEG-Cz (β = 0.51) and not by nirHEG-FpZ (β = 0.55).

In the structural part of the model, Activation left cortex significantly and positively explained TOVA variability (γ = 0.78), TOVA Commissions (γ = 0.67) and TOVA D prime (γ = 0.53). Also, as predicted, Activation central cortex positively and significantly influenced both TOVA Omissions (γ = 0.87) and TOVA response time (γ = 0.75). Moreover, like hypothesized TOVA IGCE was significantly and positively explained by TOVA variability (β = 0.11), TOVA response time (β = 0.19) and TOVA D prime (β = 0.71). Lastly, as a consequence of the re-specification of the initial model, a direct negative relation between TOVA Commissions and TOVA response time was found (β = -0.39).

Due to the goodness-of-fit and the confirmation of our predictions, this model is considered adequate to explain the relations of the data matrix. Nevertheless, as the initial model had been modified (freeing a parameter), and some of the initial hypotheses had not been confirmed, we decided to specifically test this model with the sample of subjects with ADHD to verify the results obtained.

### Multi-Group Analysis

Multi-group analysis was carried out as a cross-validation strategy to verify whether a model that has been re-specified in one sample (without ADHD) can be replicated in a second independent sample (with ADHD), which was the key aim of this study. Specifically, we used an invariance-testing strategy to test the replicability of structural paths across groups.

In the above analysis, assuming that the unconstrained model is similar in both groups, the results showed statistically significant differences concerning the five criteria examined (**Table 4**). However, no statistically significant differences were found to structural weights, [χ^2^(3) = 6.411, *p* = 0.093, NFI = 0.002, IFI = 0.002, RFI = -0.001, TLI = -0.001]. Moreover, assuming the absence of differences in structural weights, no statistically significant differences were found in structural co-variances, structural residuals, and in measurement residuals.

**Table 4:** Nested model comparison (assuming model unconstrained correct).

	^1^*MW*	^2^*SW*	^3^*SC*	^4^*SR*	^5^*MR*
χ^2^	45.104	51.515	93.428	133.575	510.189
*Df*	7	10	13	14	28
*P*	0.000	0.000	0.000	0.000	0.000
NFI	0.012	0.013	0.024	0.034	0.131
IFI	0.012	0.013	0.024	0.035	0.133
RFI	0.009	0.008	0.019	0.032	0.121
TLI	0.010	0.008	0.020	0.032	0.124

^1^*Measurement Weights,*^2^*Structural Weights,*^3^*Structural Covariance,*^4^*Structural Residuals,*^5^*Measurement Residuals*.

However, as these data revealed the equality of the models between samples taken as a whole, we determined the extent to which the model is invariant in all its parameters. Summing up, the results obtained were cross-validated and thus indicated that the re-specified model of the sample without ADHD was replicated in an independent sample (with ADHD).

### Testing the Previous Goodness-of-Fit Model in ADHD Group

In the ADHD Group, the goodness-of-fit of the hypothesized model was not adequate [χ^2^(27) = 98.684; χ^2^*/df* = 3.655; *p* = 0.000; GFI = 0.931; AGFI = 0.860; CFI = 0.973; TLI = 0.954; RMSEA = 0.102(0.081–0.124), *p ≤* 0.001]. Considering the criteria used to judge the goodness-of-fit indices, the RMSEA index revealed that the previous model did not optimally represent the relationships observed in the empirical data matrix. After examining the co-variance matrix and the modification indices, we considered including (in our model) the direct effect of the latent variable *Activation central cortex* on TOVA and D Prime. From a theoretical perspective, the inclusion of this effect seemed to be logical, because D prime is a measure of the quality of concentration obtained from the total number of omission and commission errors. Also, the central cortex area allows which is affected in students with ADHD reflected in a lower quality of the concentration given the higher number of errors. As well as eliminate indirect effect between TOVA commissions and TOVA response time (with a not significant effect *p* = 0.251). This relationship can be found in students without ADHD, but not in students with ADHD. It is because commissions are related to impulsivity, and RT is related to inattention. Thus, when both variables (impulsivity and RT) are affected, these variables can be clearly distinguished.

#### Re-specification of the Model

**FIGURE 3:**
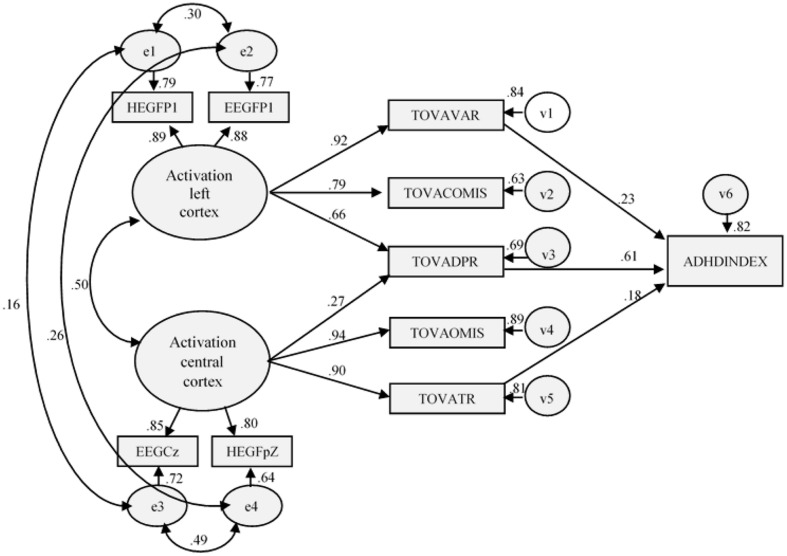
**Final model (ADHD Group): structural and measurement coefficients**.

Like inControl Group, statistically and theoretically it seemed appropriate to slightly modify the initial model in the ADHD sample by including the direct effect *Activation central cortex* on TOVA and D Prime, and thus eliminate one indirect effect. With this minimal change, the results indicated that the fit of the re-specified model was good, [χ2(27) = 98.684; χ^2^/df = 2.476; *p* ≤ 0.001; GFI = 0.952; AGFI = 0.902; CFI = 0.985; TLI = 0.975; RMSEA = 0.076 (0.053–0.099), *p* = 0.031], and also that the improvement over the initial model was statistically significant [Δχ^2^(1) = 31.820]. As expected, this newly estimated parameter was found to be statistically significant and positive (*r* = 0.27). Neither the residuals, nor the modification indices, indicated that any further changes to the model were necessary (see **Figure 3**).

The results are presented in **Table 3**. In both samples, the estimated parameters approximated the expected magnitudes and sign, and the standard errors were neither excessively large nor small. In the control Group, with the exception χ^2^ and its associated probability, the fit-indices indicated that the hypothesized model optimally represented the relationships of in the empirical data matrix. However, the data concerning fit were somewhat lower than in the first analysis. For example, χ^2^ was higher than the value of the calibration sample [e.g., Δχ^2^(1) = 40.76, and the χ^2^/df ratio rose from 2.145 to 2.476]. **Table 5** shows the coefficients of the relationships in the measurement and structural models, as well as their corresponding estimation errors, critical ratio, and associated probability.

**Table 5:** Results of testing the re-specified model in the ADHD sample).

	Standardized Coefficients	*SE*^1^	CR^2^	*P*<^3^
**Structural Model^4^**				
Activation left cortex → TOVA variability	0.918	0.045	21.557	0.001
Activation left cortex → TOVA D prime	0.662	0.005	13.364	0.001
Activation central cortex → TOVA response time	0.900	0.055	17.427	0.001
Activation central cortex → TOVA D prime	0.272	0.004	5.780	0.001
Activation left cortex → TOVA Commissions	0.794	0.055	16.459	0.001
Activation central cortex → TOVA Omissions	0.944	0.058	18.387	0.001
TOVA D prime → TOVA ADHD Index	0.608	0.091	14.251	0.001
TOVA variability → TOVA ADHD Index	0.233	0.007	5.929	0.001
TOVA response time → TOVA ADHD Index	0.179	0.006	5.601	0.001
**Measurement Model^5^**				
Activation left cortex → nirHEG-Fp1	0.889	-	-	-
Activation left cortex → Q-EEG-Fp1	0.877	0.000	25.201	0.000
Activation central cortex → nirHEG-FpZ	0.803	-	-	-
Activation central cortex → Q-EEG-Cz	0.847	0.000	23.173	0.000

^1^*Standardized errors,*^2^*Critical ratio,*^3^*Probability,*^4^*structural model (relation between the independent and the dependent variables in the model)*, ^5^*measurement model (relation between the latent variables in the model and the observed variables)*.

With regard to the predictions of the model, the results obtained in ADHD model are higher than without ADHD sample, except that the relationship between TOVA and IGCE was significantly and positively explained by TOVA variability (β = 0.23), TOVA response time (β = 0.18) and TOVA D prime (β = 0.61). Globally there were small variations that were higher in than the magnitude of the statistics obtained. *Activation left cortex* significantly and positively explained TOVA variability (γ = 0.92), TOVA Commissions (γ = 0.79), and TOVA D prime (γ = 0.66). *Activation central cortex* also positively and significantly explained both TOVA Omissions (γ = 0.94) and TOVA response time (γ = 0.90), both of which are related to attention and concentration. Lastly, as a likely consequence of the re-specification of the *with ADHD* model, a relationship between TOVA Commissions and TOVA RT was not found.

## Discussion and Conclusion

The current research attempted to deepen our knowledge of the relationship between *activation* and *executive function* measures, by examining the relationship between brain activation in selected areas and differences in executive measures. To achieve this aim we employed SEM measures, which also included latent variables such as left and central cortex activation. Although previous studies have analyzed the relationship between activation and execution, SEM has seldom been used in the past. In general, the results showed a different model for ADHD group and control group. So, one conclusion of the study is the presence of a model in which is related in a particular way, the activation in specific areas and the profile of execution of students with ADHD.

### Relationship of the Variables in the Model

In general, the data provided by the fit of the model (both in the ADHD and Control groups) provided evidence supporting some of the hypotheses proposed in the model. Therefore, the findings of this study appear to agree with those obtained in previous studies based on more conventional strategies of data analysis, such as hierarchical regression analysis and analysis of variance. The major findings discussed below concern the relationship between activation and execution, and the difference between the ADHD model and the Control model (Arns et al., 2009; Cubillo et al., 2012).

In this study, it was especially noteworthy that the relationship between activation (central and left prefrontal) and execution was stronger in ADHD subjects than in the control group. The explanation could be that subjects with ADHD show lower cortical activation (Lansbergen et al., 2011; González-Castro et al., 2013) and blood oxygenation with scores ranging between 0.38 and 0.41 for electrical activation, and between 65 and 80 for blood oxygenation, the latter of which directly affects performance patterns (in small ranges between 40 and 80). The activation levels of the control group were found to be within normal limits, however, they showed greater variations (e.g., scores ranged from 0.51 to 0.99 for electrical activation, and from 86 to 120 for blood oxygenation). All of that can be reflected in different executive patterns (large ranges of scores ranging between 85 and 120). This finding highlights the importance of analyzing electrical activation and/or blood oxygenation in the cortex. Since it is an issue that is directly related to the executive function of the subject.

Moreover, the relationship between cortical activation and executive function shows differential results depending on the brain area assessed (i.e., a low activation in a specific area can be related to a particular pattern of execution). Regarding left cortical activation, is highlighted the results indicated that differing beta-theta ratios and low blood oxygenation in area Fp1 can be related to hyperactivity and impulsivity symptomatology.

Furthermore, when the electric activation shows low levels in Fp1, these data are also supported by nirHEG results and a low performance in TOVA tests. Similarly, when the electrical activation is within normal ranges blood oxygenation and TOVA test results are also normal. While these results have been observed in previous studies analyzing the relationship between Q-EEG and TOVA, and between nirHEG and TOVA (González-Castro et al., 2013), the present research was focused on the relationships of all electrical-activation variables through a latent variable.

On the other hand, in the case of central activation, this relationship shows lower rates, and although it is observed that those who present low activation levels measured by the beta-theta ratio in Cz, also present a low oxygenation measured by nirHEG in FpZ, as well as a greater number of omission errors and worst response time; the findings do not reach so high interaction as the previous case. In any case, it has to be emphasized that being different points (Cz/FpZ), is logical that correlations decrease slightly in spite of still showing a significant relationship. Furthermore, it is possible that FpZ is also influenced by other variables besides inattention, such as emotion or anxiety control, that many studies have located in Fp2.

Firstly, given these results, the relationship between activation and execution seems to be a reliable measure for ADHD symptoms. Secondly, with regard to the differences between models from ADHD group and the control group, could be necessary its incorporation into assessment protocols in order to achieve more reliable and accurate diagnosis. Control group model shows a relationship between commissions and RT. In this sense, it is expected that an increasing of the number of commissions leads, in turn, to a low response time. By contrast, in the case of ADHD, the presence of a high commissions do not lead to a lower RT levels, since this student group also present a deficit in this variable (Leth-Steensen et al., 2000).

In the ADHD Group model, it can be observed a relationship between central activation of the cortex and D prime variable offered by TOVA. This fact makes sense, because D prime variable is obtained from the number of omissions and commission errors. Both are produced by a low level of activation in central cortical and left prefrontal brain areas. In this way, ADHD Group showed a greater number of errors both by omission and commission. Nevertheless, subjects from control group do not make omission errors, at least not significantly (González-Castro et al., 2013). Finally, comparation of both models showed differences between central and prefrontal activation relationship. While in the Control Group this relationship is 0.67, in the ADHD group decreases to 0.50. In this sense, in children without ADHD there is a relationship between different brain areas. But in the case of ADHD, the alteration in the cortical activation might present only in a specific area (Hart et al., 2013). This aspect has relevance for ADHD assessment, supported the idea about the alteration in the cortical activation and its measured through both electrical activity and blood oxygenation (Toomim and Carmen, 2009). Likewise, it is also relevant for intervention, since an improvement in the symptomatology would pass by an increase in the activation levels in the area which specifically is found more altered (Duric et al., 2014; Holtmann et al., 2014; González-Castro et al., 2016). This would imply a significant improvement because as has been reflected in this study, low activation levels in a specific area (central or left prefrontal) is particularly related to an executive profile (inattentive or impulsive/hyperactive).

### Implications for Practice

Our results have important implications in ADHD diagnosis. An Activation-Executive diagnosis model was tested to improve the assessment process in ADHD, also explained variables interactions. Moreover, this study lends support to prior studies stating that the prefrontal area is essential in ADHD assessment (Rubia et al., 2011). This leads to a model of activation in which the central prefrontal and left prefrontal areas present lower activation in children with ADHD compared to controls (González-Castro et al., 2013). These results suggest the importance of including different measures for the symptoms analysis with the aim to stablish a specific intervention and differentiate those cases that may need pharmacological support, or other interventions such as behavior therapy, neurofeedback or combine treatment. In this sense, the analysis of the activation allows professionals to determine the severity of the disorder and the intervention required.

### Limitations of the Study

Although the present study has produced interesting results, the implications derived from them should be taken cautiously as some theoretical and methodological limitations can be pointed out.

Firstly, it would have been convenient to compare the results obtained by these tests with those provided by other empirically validated tests as SPECT or fMRI, in order to compare the levels of cortical activation through blood flow and their correlations with the values provided by the HEG. Secondly, in future research, it would be appropriate to consider not only the differences between controls and ADHD subjects, but also between the subtypes of the disorder (which could reveal that different activation and execution models are needed). It would be desirable control variables and problems related to ADHD (such as anxiety or depression) which could affect the obtained results (Rodríguez et al., 2014) and specially, taking into account that the presence of a pure ADHD group is an infrequent situation. Finally, we have to note the broad age range of the sample as another limitation and highlight the interest of analyzing these measures as function of age.

## Author Contributions

CR, PG-C, MC, DA, and JG-P: Substantial contributions to the conception or design of the work; or the acquisition, analysis, or interpretation of data for the work. Drafting the work or revising it critically for important intellectual content. Final approval of the version to be published. Agreement to be accountable for all aspects of the work in ensuring that questions related to the accuracy or integrity of any part of the work are appropriately investigated and resolved.

## Conflict of Interest Statement

The authors declare that the research was conducted in the absence of any commercial or financial relationships that could be construed as a potential conflict of interest.

## Footnotes

1 http://www.biocompresearch.org
